# Systemic lupus erythematosus: a rare clinical image

**DOI:** 10.11604/pamj.2022.42.191.35508

**Published:** 2022-07-08

**Authors:** Tejaswee Lohakare, Kavita Gomase

**Affiliations:** 1 Department of Child Health Nursing, Smt Radhikabai Meghe Memorial College of Nursing, Datta Meghe Institute of Medical Sciences, Sawangi (Meghe), Wardha, Maharashtra, India,; 2 Department of Obstetrics and Gynaecology, Smt Radhikabai Meghe Memorial College of Nursing, Datta Meghe Institute of Medical Sciences, Sawangi (Meghe), Wardha, Maharashtra, India

**Keywords:** Systemic lupus erythematosus, autoimmune disease, mucosal bleeding, joint pain, photosensitive rash

## Image in medicine

Systemic lupus erythematosus (SLE), is the most common type of lupus. Systemic lupus erythematosus is an autoimmune disease in which the immune system attacks its tissues, causing widespread inflammation and tissue damage in the affected organs. It can affect the joints, skin, brain, lungs, kidneys, and blood vessels. A 12-year-girl visited the outpatient department with the complaint of skin lesions (red color) over sun-exposed areas on the hand, feet, and face. Oral mucosal bleeding, joint pain including knee, shoulder, elbow, and wrist. On physical examination, photosensitive rash and oral bleeding were observed. Laboratories investigation revealed the patient had systemic lupus erythematosus. The patient was referred to the dermatology department for further medical management.

**Figure 1:**
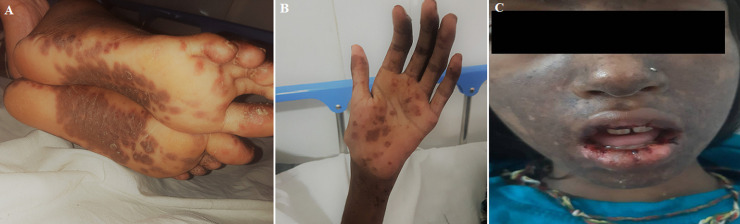
A) erythematous lesions over feet; B) erythematous lesions on palm; C) erythematous lesions over face and bleeding from oral mucosa

